# Computational methods for metastasis detection in lymph nodes and characterization of the metastasis-free lymph node microarchitecture: A systematic-narrative hybrid review

**DOI:** 10.1016/j.jpi.2024.100367

**Published:** 2024-02-04

**Authors:** Elzbieta Budginaite, Derek R. Magee, Maximilian Kloft, Henry C. Woodruff, Heike I. Grabsch

**Affiliations:** a Department of Pathology, GROW - Research Institute for Oncology and Reproduction, Maastricht University Medical Center+, Maastricht, The Netherlands; b School of Computing, University of Leeds, Leeds, UK; c Department of Internal Medicine, Justus-Liebig-University, Giessen, Germany; d Department of Precision Medicine, GROW - Research Institute for Oncology and Reproduction, Maastricht University Medical Center+, Maastricht, The Netherlands; e Pathology and Data Analytics, Leeds Institute of Medical Research at St James’s, University of Leeds, Leeds, UK

**Keywords:** Lymph node, Artificial intelligence, Segmentation, Immunity, Review

## Abstract

**Background:**

Histological examination of tumor draining lymph nodes (LNs) plays a vital role in cancer staging and prognostication. However, as soon as a LN is classed as metastasis-free, no further investigation will be performed and thus, potentially clinically relevant information detectable in tumor-free LNs is currently not captured.

**Objective:**

To systematically study and critically assess methods for the analysis of digitized histological LN images described in published research.

**Methods:**

A systematic search was conducted in several public databases up to December 2023 using relevant search terms. Studies using brightfield light microscopy images of hematoxylin and eosin or immunohistochemically stained LN tissue sections aiming to detect and/or segment LNs, their compartments or metastatic tumor using artificial intelligence (AI) were included. Dataset, AI methodology, cancer type, and study objective were compared between articles.

**Results:**

A total of 7201 articles were collected and 73 articles remained for detailed analyses after article screening. Of the remaining articles, 86% aimed at LN metastasis identification, 8% aimed at LN compartment segmentation, and remaining focused on LN contouring. Furthermore, 78% of articles used patch classification and 22% used pixel segmentation models for analyses. Five out of six studies (83%) of metastasis-free LNs were performed on publicly unavailable datasets, making quantitative article comparison impossible.

**Conclusions:**

Multi-scale models mimicking multiple microscopy zooms show promise for computational LN analysis. Large-scale datasets are needed to establish the clinical relevance of analyzing metastasis-free LN in detail. Further research is needed to identify clinically interpretable metrics for LN compartment characterization.

## Highlights

• Structural changes in metastasis-free lymph nodes show clinical importance.
• Artificial intelligence could facilitate the analysis of lymph node images.
• Multi-scale deep learning models are frequently chosen for pathology tasks.
• Further research is warranted for clinically interpretable lymph node characterization.

## Graphical abstract

**Figure:**
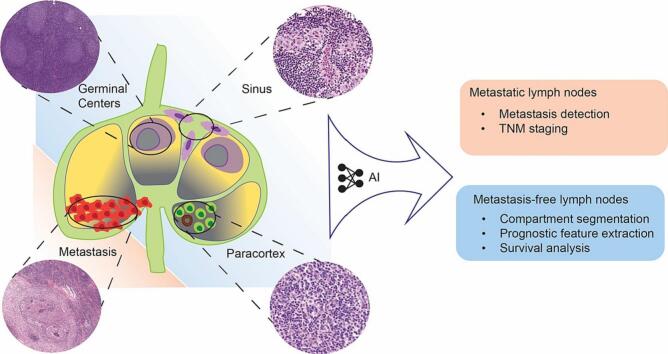

## Introduction

The histological examination of lymph nodes (LNs) is an essential part of cancer staging. The LN status (presence or absence of metastasis) is considered a key prognostic factor in patients with various different cancer types, including breast, gastric, esophageal, and colorectal.^1^, ^2^, ^3^, ^4^ The number of regional LNs with metastases as well as the extent (size) of the metastatic lesion within each LN, which may vary from a macroscopically visible lesion to a micro-lesion consisting of a few isolated tumor cells, are the main characteristics defining the regional spread of the cancer.^5^

**Fig. 1:**
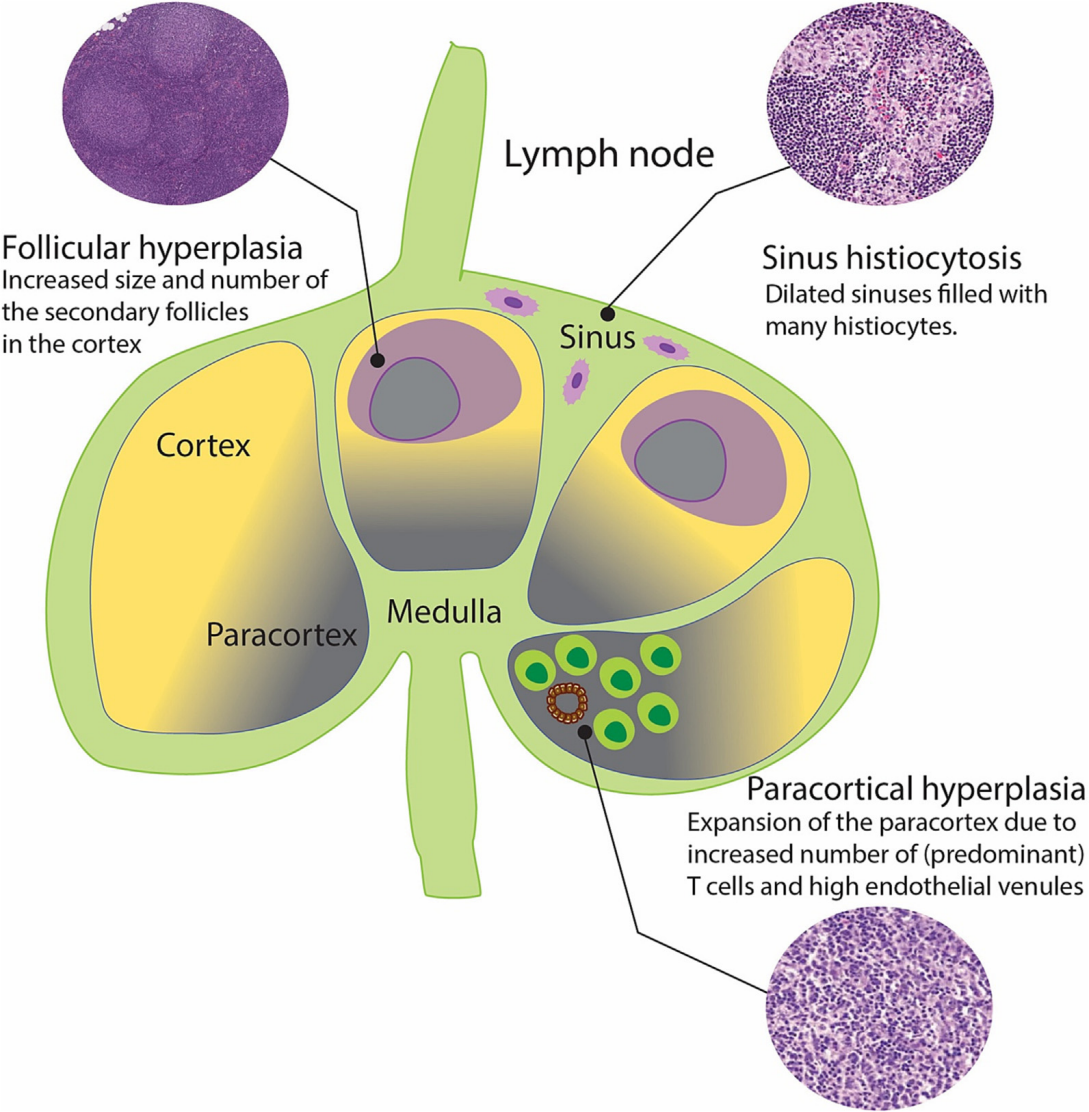
Schematic representation of immunologically stimulated lymph node with enlisted compartment-specific reactive patterns.

There is evidence accumulating which suggests that metastasis-free LN microarchitecture rearrangements might be clinically important.^6^, ^7^, ^8^ The LN microarchitecture can be divided into distinct layers exhibiting different compositions and functions, see Fig. 1. The outer layer, named cortex, consists of primary follicles composed of naïve B-cells. Upon immune stimulation, these primary follicles can transform into secondary follicles with germinal centers where further B-cells proliferate. The next (deeper) LN layer, named paracortex, typically contains T-cells and dendritic cells which enter the LN via high endothelial venules (HEVs). The innermost layer of LN, called medulla, containing plasma cells, B-cells and macrophages, opens into hilum and efferent lymphatic vessels. The LN has a conduit system (so called sinuses) that transports macrophages and antigens from outside the LN to the inside. Changes in the LN can manifest as so-called reactive patterns which are illustrated in Fig. 1. The presence of structural changes such as sinus histiocytosis has been associated with the presence of a host anti-tumor response in patients with breast, gastric, and colorectal cancers.^9^, ^10^, ^11^ In the same cancers, the presence of LNs with follicular hyperplasia were related to a longer patient survival.^12^, ^13^, ^14^ It has been suggested that the presence of a hyperplastic LN paracortex might be a predictor of better survival in gastric cancer patients.^15^ It has also been suggested that HEVs, usually present in the LN paracortex, may undergo morphological remodeling in pre-metastatic LNs and hence could be a potential morphological biomarker for prediction and prognosis in cancer patients.^16^

**Fig. 2:**
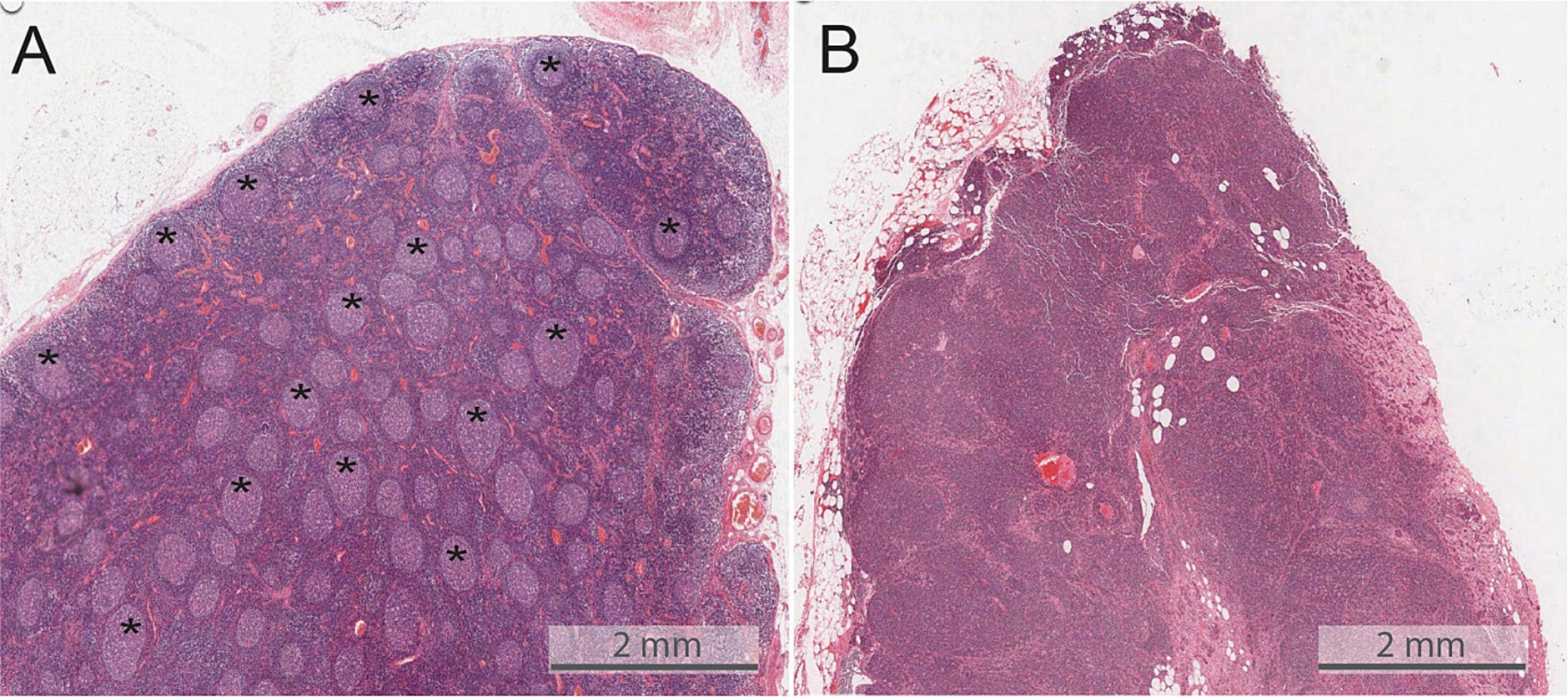
Exemplary images taken from H&E stained lymph node from esophageal cancer patients. The figure illustrates the visually perceivable differences between immunologically stimulated (also called reactive) LNs. Figure A shows a lymph node with a large number of germinal centers (some are highlighted in asterisk), indicative of follicular hyperplasia; Figure B contains a metastatic lymph node with hyperplasia of paracortex and a lack of germinal centers.

To validate the clinical importance of reactive LN patterns or reactive LNs as a whole, large scale investigations need to be performed on sufficiently large cohorts. In order to obtain the measurements of individual LN compartments, these would need to be segmented manually which is very time consuming. Thus, in order to generate large-scale evidence, there is a need for automated LN compartment analysis methods. As LN reaction patterns can be seen through a microscope (see Fig. 2), digital histological images containing LNs should be suitable for artificial intelligence (AI)-assisted analysis.

The launch of the Camelyon16 challenge in 2016 marked the start of a very productive period for researchers investigating computational methods for metastatic LN tumor characterization. The Camelyon datasets consist of 1399 digitized axillary LN slides with partial metastatic tumor annotations and patient-level pathological lymph node (pN) status.^17^ The slides from Camelyon datasets were collected from multiple centers and thus vary in stain intensities and sample preparation protocols, reflecting the practical challenges facing AI models such as generalizing across multi-center data. Since the announcement of the Camelyon challenges,^17^ there has been a relatively large number of publications proposing different algorithms to detect LN metastases using the publicly available whole slide images (WSI) of axillary LNs from breast cancer patients. However, the computational characterization of metastasis-free LNs seems to be lagging behind, perhaps due to the fact that there are no well-established benchmarks like the Camelyon challenge and no publicly available annotated datasets. This review aims to systematically identify and analyze recent publications that applied some form of AI for LN characterization, focusing on the identification of AI methodologies in LN analysis, clinical interpretability of the AI-based results, and current shortcomings of AI tools potentially impeding clinical implementation.

## Methods

### Search strategy

This review was conducted following the proposed reporting items for systematic reviews and meta-analyses (PRISMA) protocol.^18^ We searched for publications across the following databases: PubMed, Scopus, ArXiv, Web of Science, and Institute of Electrical and Electronics Engineers Xplore, and the search phrases are listed in the supplementary document S1. The databases were searched for articles published up to December 1, 2023.

### Selection criteria

Studies focusing on lymph node (LN) detection, computational LN characterization, or compartment segmentation in histological whole slide images (WSI), including hematoxylin and eosin (H&E) and immunohistochemistry (IHC) staining using AI methods, either deep learning or machine learning, were included in our analysis. Studies reporting on imaging modalities other than histopathology, such as positron emission tomography, computer tomography, ultrasound scans, tissue simulation studies, and tumor immune microenvironment studies were outside the scope of this review and were thus excluded, as well as studies published in other languages than English.

### Screening by title and abstract

To select relevant studies eligible for full text screening, the initially identified studies were filtered based on title and abstract content considering the above-mentioned inclusion/exclusion criteria.

### Full text screening

The full text of eligible articles was further analyzed to identify and exclude out of scope articles. Articles with unretrievable full text, as well as conference abstracts were excluded from further processing. Data such as dataset, AI model, cancer type, and study objective were extracted from each full text for further article assessment.

### Study quality assessment

In order to assess the experimental design of the study and risk of bias, quality assessment was performed following the Checklist for Artificial Intelligence in Medical Imaging (CLAIM) protocol.^19^ For quality assessment, a detailed description of the AI model used in the study as well as clearly defined training data were deemed mandatory for the study to be included in the current review.

## Results

**Fig. 3:**
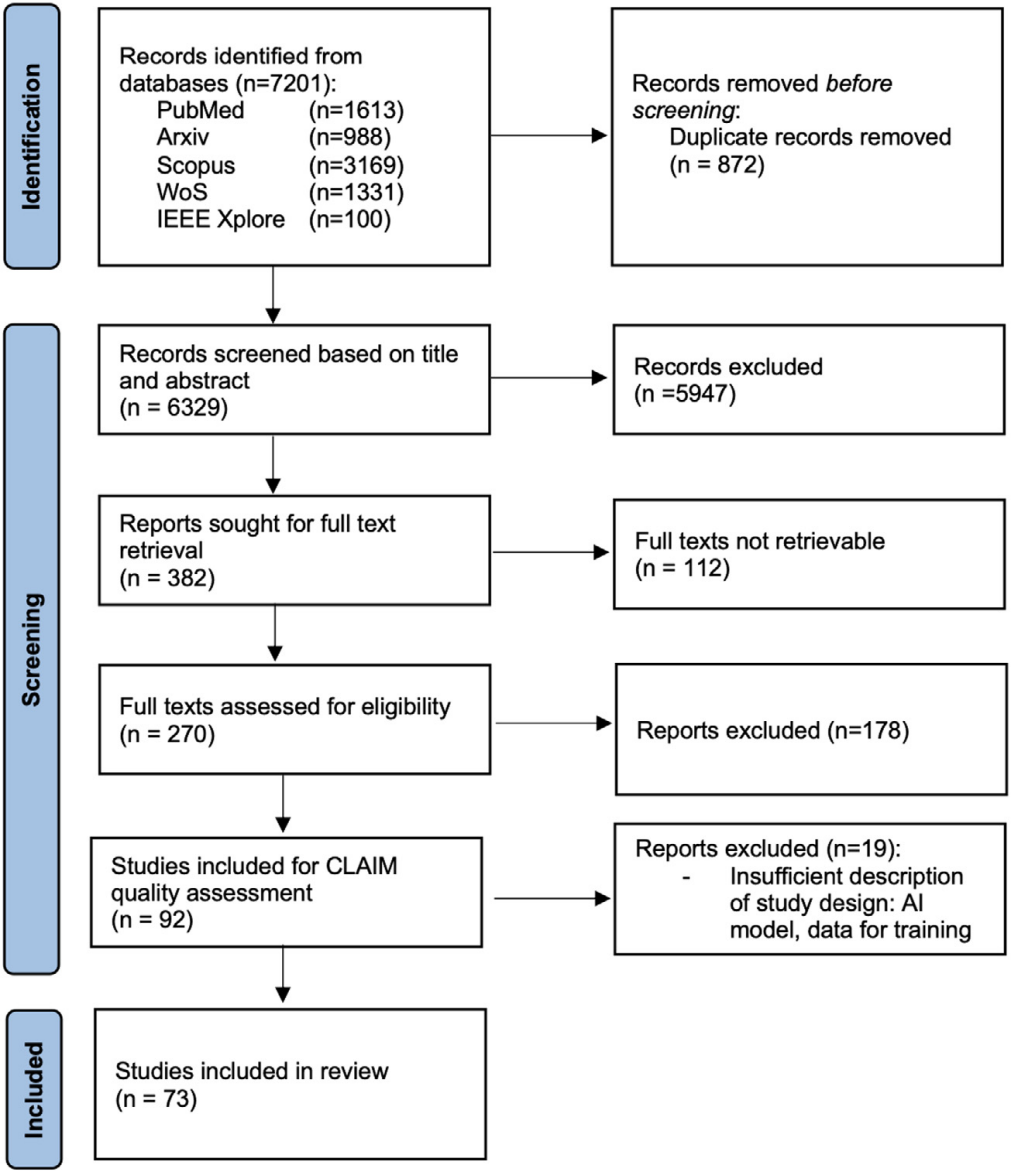
PRISMA flowchart illustrating the article screening and selection process implemented in this systematic search.

Following the inclusion and exclusion criteria, the PRISMA protocol for systematic reviews, and the CLAIM protocol for study assessment, we finally identified 73 studies to be included in our systematic review, see Fig 3 for PRISMA flowchart and Supplementary Table S2 for CLAIM assessment results.

### Datasets

56% (*n*=41) of the studies included in this review utilized publicly available datasets (see Supplementary table S3). 36% (*n*=26) of the studies used the Camelyon16 dataset,^20^ consisting of 399 H&E stained breast cancer sentinel lymph node (SLN) WSIs with LN status labels (with metastases versus without metastases) and partial pixel annotations of metastatic lesions. The second most widely utilized dataset was Patch Camelyon,^21^ which was used by 8 (11%) studies and which is a derivative patch version of Camelyon16 (see Table 1). Eight (11%) studies used Camelyon17,^17^ a multi-center dataset extending Camelyon16 to 1399 breast cancer SLN WSI. Two (3%) studies utilized the SLN breast cancer dataset from the cancer imaging archive (TCIA) with slide based LN status labels.^22^ One study reported results obtained from an axillary LN breast cancer dataset available from the Analytic Image Diagnostics Arena (AIDA) Data Hub, which provides slides immunohistochemically stained for cytokeratin (clone AE1/AE3) instead of manual annotations as ground truth for cancer metastasis.^23^ There were two publicly available LN datasets that did not focus on metastasis detection. The dataset published by Bekkhus et al^16^ was used for HEV segmentation in immunofluorescently stained LN slides from breast cancer including metastatic and metastasis-free LNs. Furthermore, Gamez Serna et al published a dataset with their multi-magnification organ network (MMO-Net) that included rat mandibular LNs with annotated LN contours.^24^ More details on each dataset are provided in Table 1.

**Table 1:** Summary of publicly available datasets containing LN tissue sections.

Dataset	Summary	Ground truth	Sample size	Dimensionality	Suitable applications
Camelyon16	H&E stained slides of breast cancer sentinel lymph nodes from 2 centers	Patient-level labels: pN stage. Pixel-level: partial tumor annotations	399 WSIs	WSIs scanned at 20x and 40x magnifications	Segmentation, slide-level classification, MIL
Camelyon17	H&E stained slides of breast cancer sentinel lymph nodes from 5 centers	Patient-level pN stage label Pixel-level: partial tumor annotations	1000 WSIs	WSIs scanned at 20x and 40x	Segmentation, classification, MIL
Patch Camelyon	Patches extracted from Camelyon16 dataset	Patch-level label: tumor positive or negative	327,680 patches	96x 95 pixel patches at 10x	Patch classification
SLN-Breast, TCIA	H&E stained slides scanned in one center	Patient-level labels: pN stage label	130 slides from 78 patients	WSIs scanned at 20x	Slide-level classification, MIL
Axillary lymph nodes in breast cancer, AIDA	H&E slides of breast cancer sentinel lymph nodes (2 consecutive slices) from multiple scanners	Patient-level labels: treatment, pN stage, primary tumor. IHC slides for cytokeratin AE1/AE3	396 patients	WSIs scanned at 20x	Tumor segmentation, slide-level classification, MIL
Breast cancer lymph nodes for HEV detection	Immunofluorescently stained HEVs using HEV markers for peripheral node addressin PNAd and vascular marker Claudin-5	Immunofluorescence signal for HEVs	73 patients with invasive, non-invasive breast carcinoma, or cancer-free	Not specified	HEV segmentation and diameter evaluation in LNs
Multi-magnification organ network (MMO-Net) dataset	H&E stained slides of rat mandibular lymph nodes from 3 centers	Manual LN contour annotations	53 WSIs containing lymph nodes, 267 WSIs from other organs	Not specified	LN contour segmentation

**Fig. 4:**
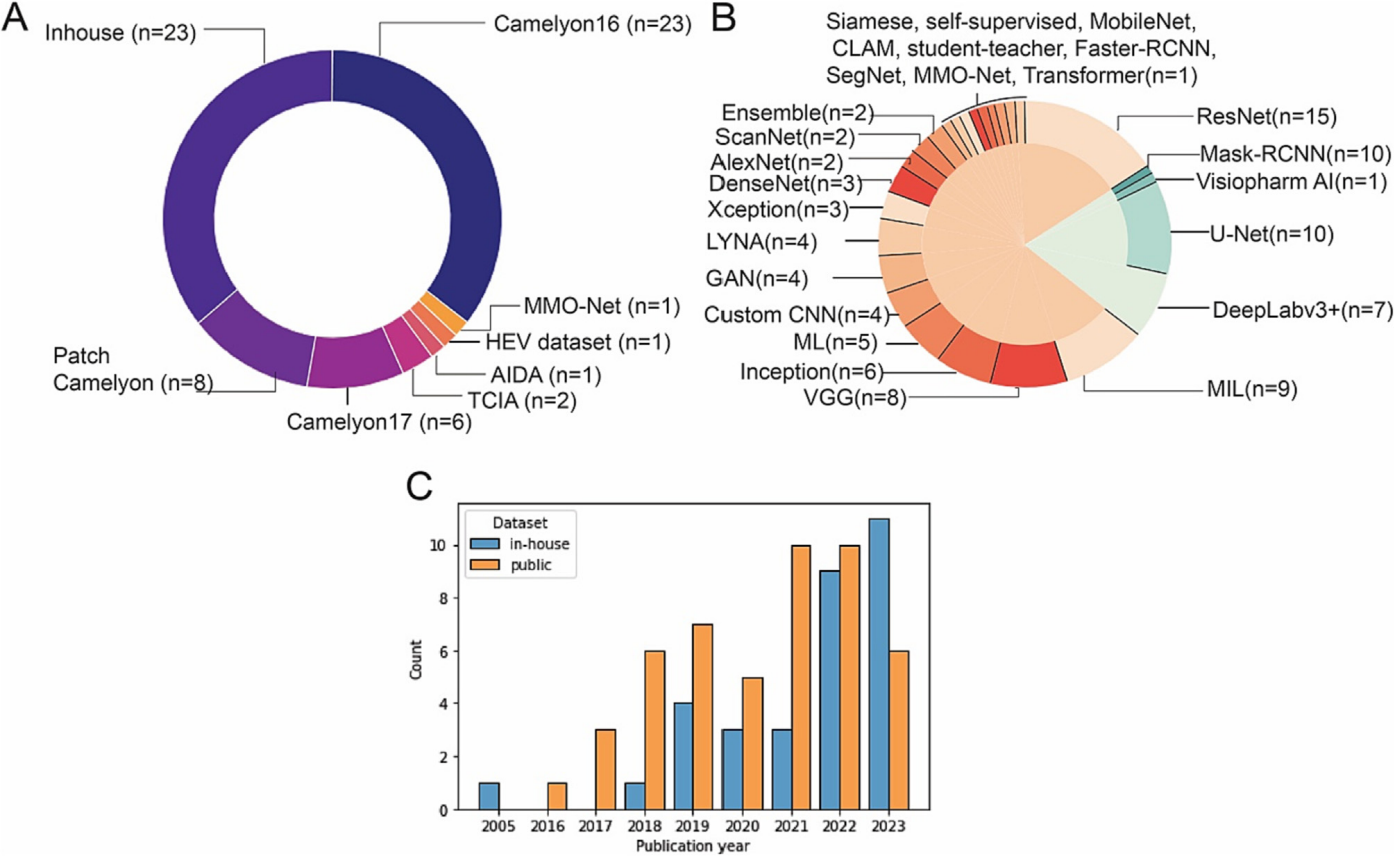
Graphs illustrating the usage patterns of the datasets and models in selected studies. A: piechart with dataset distribution across the selected papers, B: piechart illustrating the model selection in the included articles. The total number of models is larger than the number of studies, as some studies trained more than one model. C: chronological chart of dataset usage focusing on inhouse/public dataset selection. The barchart illustrates the steady increase of studies using inhouse datasets.

32 studies (44%) utilized publicly unavailable in-house datasets. The use of in-house datasets increased steadily overtime as can be seen in Fig. 4C.

### Lymph node contour detection

From an anatomical perspective, LN contour detection is the most general task in computational LN analyses. We identified eight studies proposing automated LN detection solutions. An early adoption of machine learning techniques was used in a study by Niemisto et al.^25^ In 2005, where K-means clustering algorithm was used to segment LNs based on a simplified three-color clustering scheme. Verghese et al^26^ applied a modified Otsu thresholding method to localize LNs in the whole slide breast cancer images. In the study by Wang et al,^27^ a U-Net autoencoder was trained using 1x magnification regional gastric cancer LN images to localize the LNs within the slide. A more sophisticated approach for LN segmentation was recently reported by Gamez Serna et al,^24^ where a DenseNet121-derived MMO-Net model was trained to detect rat mandibular LNs in a multi-scale fashion accounting for two different image magnifications in parallel (5 and 1.25). Huang et al^28^ trained DeepLabv3+ segmentation model for LN detection in gastric cancer patients at 1.25x magnification, whereas Hu et al^29^ utilized a pipeline of faster recurrent CNN (Faster-RCNN) and DeepLabv3+ models for LN bounding box detection followed by precise LN contour delineation. A two-step LN detection approach has also been implemented by Beuque et al,^30^ where a U-Net model was trained to obtain LN mask from WSI thumbnail, followed by false-positive prediction filtering by XGBoost model trained on hand-crafted radiomics features obtained from LN masks. Faster-RCNN model was also used for LN detection by Tan et al,^31^ where a model was trained to detect LN bounding boxes in colorectal cancer patients at 5x magnification.

### Lymph node metastasis detection

Metastatic LN status identification is essential for predicting cancer patient’s prognosis and determine further treatment. In our systematic review, 63 (86%) out of 73 studies focused on metastatic lesion detection in regional LNs. 48 out of those 62 studies reported results from analyzing metastatic breast cancer regional LNs. This bias towards LN metastasis detection in breast cancer can be largely explained by the availability of public datasets, namely Camelyon16 and Camelyon17. Based on the machine learning paradigm utilized in a study, we grouped the metastasis detection articles into six groups.

### Studies using convolutional neural network models

The use of convolutional neural networks (CNNs) has been widely adopted in AI-assisted image analysis field. The CNN architecture relies on convolutional filters called kernels—small matrices with learnable weights. The convolution process entails a sequential kernel application across the input image matrix, where a feature map is created via element-wise multiplication of the kernel and a subset of input matrix.^32^ There were multiple CNN architectures suggested throughout the years which have been universally applied in computer vision tasks. In 2014, Google has introduced their GoogleNet model, also called Inception^33^—a CNN model that suggested a parallel utilization of multiple kernels of different size. A year after ResNet50 architecture was suggested,^34^ solving the vanishing gradient issue via skip (residual) connections. The original model contained 50 layers, with alternative ResNet101 (101 layers) and ResNet152 (152 layers) versions. Vanishing gradient problem was also addressed by DenseNet model,^35^ where each layer receives an input from all prior layers via dense connections.

Out of 63 articles discussing metastatic LN analysis, 27 papers were dedicated to a classic deep learning strategy for computer vision, where one or multiple CNN architectures were trained and tested on LN metastasis detection datasets. The earliest studies included in our review starting in 2016 applied commonly used CNN architectures such as AlexNet, DenseNet, GoogleNet and Visual Geometry Group CNN (VGG), as well as custom architectures like ScanNet on Camelyon 16 dataset.^36^, ^37^, ^38^, ^39^, ^40^, ^41^ The Camelyon16 dataset was further utilized to extract metastatic and metastasis-free LN patches into a dataset called Patch Camelyon, which was applied for patch classifier models.^21^ Later Camelyon dataset usecases were adapted to address practical WSI processing challenges. WSI scanning speed was addressed by Zhang et al,^40^ where authors proposed a multiple spatial context network and patch feature sharing for faster slide scanning. Shvetsova et al^42^ proposed unsupervised anomaly detection in Camelyon16 using an autoencoder reconstruction of the input image focusing on the evaluation of the perceptual loss. Alheejawi et al^43^ proposed a deep learning method for proliferative index (PI) evaluation in LNs of melanoma cancer in Ki-67 stained WSIs. The authors used a SegNet model to obtain pixel-level segmentation masks. Hu et al^29^ utilized inhouse gastric cancer dataset to train DenseNet121 model for metastatic LN detection. Pham et al^44^ trained a pipeline of two VGG models for germinal center and metastasis detection, concluding that additional secondary follicle detection step improved tumor model specificity. Allam et al^45^ suggested a novel approach towards LN metastasis identification in breast cancer patients—instead of predicting metastasis directly from metastatic regions, authors trained a custom architecture CNN model to detect metastasis from non-metastatic LN regions (metastatic tumor microenvironment). The authors evaluated LN sinuses, follicles, and interfollicular lymphocytic areas concluding that lymphocyte-rich interfollicular regions had the highest positive predictive power for metastatic LN detection.

13 studies (18%) utilized ResNet model architecture to detect LN metastasis. Out of these studies, five articles utilized Camelyon datasets. Jaiswal et al^46^ trained classical patch classifiers such as ResNet, VGG, Inception on Patch Camelyon in semi-supervised fashion using class with maximum predicted probability as ground truth labels for unlabeled patches. The authors used test time augmentation technique to expand the test dataset and evaluate model generalizability. Kim et al^47^ used the Camelyon datasets on the Inceptionv3, VGG16, and ResNet models as transfer learning for their in-house dataset of frozen H&E breast cancer LN samples validating that the features learned on Camelyon datasets were transferable to frozen tissue samples. In a study by Chen et al,^48^ the authors used a RRCART model to distinguish high-accuracy metastasis predictions from low-accuracy predictions obtained using ResNet model. Lee and Paeng et al^49^ passed a tumor probability map generated with ResNet101 into a random forest model for pN prediction, achieving second best-weighted kappa score of 0.9203 within the pool of selected studies leveraging Camelyon17 dataset. Patil et al^50^ introduced HistoROI model—a derivative of ResNet architecture that was designed to keep the diagnostician in the loop via active learning. Turki et al^51^ trained multiple models, namely ResNet, DenseNet121, VGG16, and Xception on the SLN-breast dataset from TCIA, concluding that DenseNet121 achieved the highest performance for their dataset of choice.

Wang et al^27^ used the ResNet50 binary patch classifier for gastric cancer regional LN segmentation, using germinal center, sinuses, and adipose tissue annotations for the identification of the metastasis-free LN class. This study also analyzed the spatial distribution of metastatic lesions, specifying two distinct metastatic dissemination patterns in gastric cancer patients. Chuang et al^52^ utilized ResNet50 for metastasis identification in colorectal LNs from colorectal cancer patients coupled with class activation mapping to highlight diagnostically important regions for the model. ResNet model has been utilized for LN metastasis detection in gastric cancer patients by Huang et al^28^ and Matsushima et al^53^ who concluded that utilization of ResNet model significantly increased the micrometastasis detection sensitivity compared to manual unassisted analysis, however, the automated slide analysis took longer than time needed by pathologist. In the study by Kronberg et al,^54^ authors trained a multi-class model for metastatic LN detection in pancreatic cancer patients, capable of distinguishing between healthy pancreas, metastasis-free LN, metastasis, background, and adipose classes.

When designing an automated diagnostic tool, it is important to minimize the chance of false-negative decision, where a metastatic LN is assigned to a “healthy” metastasis-free class, which is reflected by sensitivity metric. Two studies in our analysis proposed ResNet-based solutions that achieved a perfect 1.00 sensitivity in gastric cancer^55^ and head and neck cancer^56^ datasets consisting of 40 gastric cancer patients and 50 head and neck cancer images, respectively. The proposed metastasis detection systems reached lower yet still competitive false-positivity (specificity) scores of 0.9994 in gastric cancer and 0.759 in head and neck cancer studies, respectively.

### Studies using convolutional autoencoders

Autoencoders are a specific type of CNN networks consisting of encoder and a decoder part aiming to learn a condensed representation of input image. The encoder part compresses the input image into an embedding vector in the autoencoder bottleneck, whereas a decoder part is responsible for input image reconstruction from the embedding latent space. In contrast to the previously described CNN classifier models, which assign a class to the input image patch, autoencoders can produce a finer output mask by classifying each pixel within an input image into a predefined class. In 2015, Ronnenberger et al^57^ introduced a U-Net—a U-shaped autoencoder architecture containing skip connections between encoder and decoder parts to help preserve the spatial information. Two years later Google introduced a DeepLabv3+ model.^58^ The DeepLabv3+ architecture utilizes patch classifier models such as ResNet50 or DenseNet for feature extraction followed by dilated (also called atrous) convolutions for a wider receptive field and contains an atrous spatial pyramidal pooling (ASPP) module designed for multi-scale feature extraction via multiple atrous convolution rates.

Eight studies utilized pixel-level segmentation models, namely U-Net, DeepLabv3+, or their derivatives. Xu et al^59^ leveraged a U-Net model to segment metastases in LNs of the Camelyon16 and Camelyon17 datasets followed by random forest model for pN stage prediction. Jin et al^60^ introduced ConcatNet model by concatenating four U-Net models each for nucleus, mitosis, epithelium, and tubule segmentation. Jansen et al^61^ utilized U-Net model with ResNet50 encoder for metastasis identification in LNs from melanoma patients achieving competitive sensitivity scores of 91.67 and 95.62 on two datasets. Mainovskaya et al^62^ used a pixel segmentation approach using DeepLabv3 model for metastasis segmentation in CRC LNs. The authors also considered the presence of slide artifacts such as tissue folds and pigment residues, which were addressed by training an additional U-Net model for artifact detection. DeepLabv3+ model was applied for metastasis identification in breast^63^ and esophageal^64^ cancer studies. Bozdag et al^65^ addressed the large parameter space of DeepLabv3+ model and introduced a lightweight pyramid-structured segmentation network NonLocalSeg for more time-efficient slide processing in the Camelyon16 dataset. Wang et al^66^ utilized a modified DeepLabv3+ model by introducing pyramidal attentional atrous spatial pyramidal pooling (PA-ASPP) and scale-aware selection to weight multi-scale features extracted by ASPP using ResNet101 model as feature extractor. To obtain slide-level metastasis predictions, the authors used density based spatial clustering model (DBSCAN) to combine patch-level predictions and XGBoost model for patient-level pN prediction. The proposed model framework achieved weighted kappa score of 0.9632 which was the highest score among included articles utilizing Camelyon17 dataset in this review.

### Multi-scale models

Among the selected study pool, we identified five articles that used multi-scale models. Multi-scale models, originally proposed by Wang et al in 2016,^67^ leverage tissue context information by ingesting two input images of different magnification at the same time, thus mimicking multiple microscopic zoom levels. In 2017, Liu et al presented Google’s Lymph Node Assistant (LYNA) model trained on Camemyon16 patches.^68^^,^^69^ The LYNA model was built on Inception V3 architecture in multi-scale fashion. Steiner et al^70^ reported that the use of the LYNA model as digital assistant for pathologists significantly reduced the slide screening time needed to reach a final diagnosis. The authors have also tested the LYNA framework on their in-house dataset and reported the LYNA model robustness against slide artifacts such as bubbles and H&E stain variability. In a study by Schmitz et al,^71^ the authors investigated multi-scale WSI training strategies, achieving higher weighted Jaccard coefficient values for the Camelyon16 dataset compared to the use of a single-scale U-Net model. In recently published study by Wang et al,^72^ authors have also utilized Camelyon16 dataset for their multi-scale model, that was based on ResNet architecture. The authors proposed a model consisting of low- and high magnification networks operating at 10x and 40x magnifications, respectively. The model was designed to first scan the slide at low magnification to identify regions of interest that would further be analyzed at high magnification. The proposed multi-scale model was compared to LYNA model with respect to the testing FROC metric and processing time, achieving higher FROC performance with significantly lower slide processing time (33 min per slide for LYNA model compared to 4.5 min for proposed model).

### Generative adversarial networks

To expand a particular given dataset and improve deep learning model generalizability, image augmentation is often employed, including image rotation, axis flipping, and/or color saturation variations. A more advanced method to augment the dataset is by leveraging generative adversarial networks (GANs)—a specific family of deep learning models trained to generate images from random noise—for synthetic data generation. Four studies trained GANs on the Camelyon datasets. Kovalev et al^73^ employed two GAN models, namely deep convolutional GAN and progressive growing GAN to extract deep features from the synthetic images and trained classic machine learning models for image classification into either metastatic or metastasis-free image patch. Jiarong et al^74^ introduced HistoGAN model for synthetic image generation which was further applied in a study by Xue et al^74^ proving that synthetic image generation improves classification model performance. Due to the usually site-specific sample preparation protocols, staining intensity variability, tissue thickness variability, and scanner-specific artifacts, it is challenging to build a generalized model capable of performing the task equally well using slides from different centers. Wollmann et al^75^ explored this issue by using a CycleGAN model for the domain adaptation task. The generative model was trained to transfer the medical center-specific styles to the slides from new medical centers unseen by the deep learning model aiming to reduce inter-center image variability.

### Multiple instance learning

The development of deep learning systems usually requires large amounts of data with labeled ground truth, which is a challenge in pathology field due to the limited time resources of pathologists that annotate the data and large WSI scale. The large WSI dimensions (on average, 100.000 x  100.000 pixels)^76^ complicate the annotation process, rendering the full WSI manual annotations a barely realistic option. Camelyon datasets are a good example of annotation sparsity, where only partial pixel annotations are available accompanied with a patient-level label. The challenges of sparse data annotations can be facilitated by applying multiple instance learning (MIL) paradigm. MIL training is a branch of weakly supervised model training approaches, where the training instances are bagged in batches with a single label per bag. For our analysis, we identified nine articles implementing this approach, eight of which leveraged Camelyon datasets. Akbar et al^77^ implemented a MIL model for Camelyon16 dataset by training a variational autoencoder in unsupervised manner and subsequently clustering the learned features into pre-defined labels. A categorical cross-entropy loss modification was introduced for weak label assignment. Courtiol et al^41^ implemented MIL via learning the top instances and negative evidence. Shao et al^78^ utilized visual transformer model for transMIL approach. The same authors produced attention heatmaps to visualize the image areas deemed important by the AI algorithm, rendering the result more explainable. Wang et al introduced a second-order MIL relying on the matrix power normalized covariance pooling, so-called second-order feature extraction, from LN slides. The authors applied a channel attention mechanism to identify the most discriminative second order features in the slide. Wang et al^79^ introduced a label cleaning MIL approach for sparse annotation refinement using only single WSI for model training, thus suggesting an alternative to the regular data-intensive deep learning training protocols. Kang et al^80^ used transfer learning technique for their MIL approach by training the model jointly on Camelyon dataset and their in-house esophageal cancer dataset. The use of publicly available dataset reduced the need for high-quality inhouse annotations. Sadafi et al^81^ implemented attention-based MIL approach with active learning. The proposed system evaluated the uncertainty of classification results submitting the slides with the least certain predictions to the expert for additional annotations. Yu et al^82^ proposed vocabulary-based MIL approach, where the model was trained to discover the structural prototypes metastatic LNs from breast cancer patients via unsupervised clustering, thus offering a higher model interpretability. Tan et al^31^ proposed a transformer-based MIL model for colorectal cancer cases. The authors emphasized that the attention map obtained with the transformer model correctly localized the metastatic LN lesions even if the final prediction was negative, suggesting the model’s capability of avoiding false negatives. Transformer model has also been utilized by Qin et al,^83^ achieving a higher sensitivity in Camelyon16 dataset compared to pathologist (94.25% and 73.2%, respectively).

### Other machine learning methodologies

In the field of computer assisted diagnosis, error rate minimization is of paramount importance. One way to reduce the variance of predicted model errors is to use a combination of multiple machine learning models to reach the final prediction—a method called model ensembles. In the pool of our selected papers, there were two papers leveraging this approach for metastasis detection. Abdollahi et al^84^ and Munappa et al^85^ ensembled models such as ResNet, DenseNet, and VGG on Camelyon datasets, Munnappa et al reported that ensemble model achieved higher precision, recall, and F1 score compared to single models. The speed of WSI processing was increased using reinforcement learning (RL) as suggested by Zhao et al.^86^ The authors proposed the RLogist model, which tries to mimic the histopathologist’s real-world approach in finding the regions of interest and analyzing those regions in multi-scale fashion. Gildenblat et al^87^ investigated a self-supervised deep learning model, training a Siamese network. The authors leveraged the spatial continuity, treating adjacent tiles as more similar than distant ones, yielding a better tumor tile retrieval ratio compared to ordinary self-supervised models. A teacher–student model paradigm was investigated in a study by Cheng et al,^88^ where the spatial distance was leveraged as tile similarity metric. Shubin et al^89^ suggested including the variance error into deep learning model loss function, assuming that the variance error is included in the bias-variance trade-off. Challa et al^90^ utilized a commercial software for LN metastasis detection offered by Visiopharm, achieving 100% sensitivity and relatively low specificity score of 41.5% in their breast cancer LN datasets.

Finally, there was a small percentage of studies (*n*=3, 4%) describing non-deep learning AI methods for metastasis identification. Valkonen et al^91^ compared patch classification task performance between a neural network and classic machine learning algorithms, such as support vector machine and random forest. Palatnik de Sousa et al^92^ explored the explainability of the Camelyon16 dataset classification using locally interpretable model-agnostic explanations technique for Patch Camelyon patches split into superpixels by the SLIC algorithm. The follow-up study by the same authors^93^ proposed genetic algorithm-inspired evolved explanation (EvEx) methodology to contrive model explanations and extract segmentation masks based on the explanation results.

### Interpretation of LN metastasis misclassification

Only 12 studies (19%) out of 63 studies investigating LN metastasis provided a detailed analysis of false-positive predictions in metastatic LN detection. Based on the gathered data, LN sinus histiocytes were the most common false positively identified region mentioned by six studies^29^^,^^31^^,^^56^^,^^68^^,^^90^^,^^94^ followed by secondary lymphoid follicles (germinal centers),^56^^,^^64^^,^^94^ connective tissue,^31^^,^^64^ out-of-focus areas,^68^ and slide artifacts.^50^ The most common metastatic regions misclassified as negative were micrometastatic lesions^61^^,^^64^ as well as histiocyte-like tumor cells.^53^

Wang et al^72^ identified adipose tissue, out-of-focus regions, and tumor edges as the areas where their model was struggling the most. To reduce false-positive rates for metastatic lesions, Pham et al^44^ used a two-step deep learning pipeline including a follicle class to the metastasis detection model pipeline.

### Segmentation of lymph node compartments

For this review, we found six (8%) articles focused on metastasis-free LN tissue characterization. Bekkhus et al^16^ proposed a Mask-RCNN model for HEV segmentation in immunofluorescent stained LN samples called HEVfinder aiming at identification of dilated HEVs in tumor draining LNs in breast cancer. One study analyzed the clinical significance of extracellular matrix remodeling in metastatic LNs. Qaiser et al^95^ studied tumor proximity to immunohistochemically detected collagen IV fibers in diffuse large B-cell lymphoma. The study utilized the HydraNet model to detect tumor cells and k-means clustering to identify the association strength between tumor cells and collagen regions in metastatic LNs. Kurian et al^96^ proposed a U-Net derived models for LN sinus and germinal center segmentation implementing multi-scale and confidence map features into their deep learning solution. The authors implemented fuzzy boundaries to tackle imperfect sinus edge segmentations. A recent study by Jin et al^97^ aimed for detailed LN computational representation using 11 classes for LN segmentation. The study reused the patch-based classification approach using the LYNA model. This study aimed at real-time LN compartment predictions for augmented reality microscopy. The authors raised concerns about the choice of the optimal magnification for the model, illustrating this with tumor cell detection differences at 20x and 40x magnification. Verghese et al^26^ trained multi-scale U-Net autoencoders with atrous convolution layers for LN sinus and germinal center detection in H&E-stained breast cancer slides. The authors trained the model using WSI crops at 2.5x, 5x and 10x magnifications, concluding that a mixture of different slide magnifications during training improved the final U-Net model performance. Song et al^98^ utilized ResNet model to classify breast cancer LNs into either obese, metastatic, or metastasis-free achieving 0.67 AUC classification score. The slide tiles deemed the most representative by the model were further processed via rule base filtering to quantify adipocytes, erythrocytes, and lymphoid white space leading to a conclusion that increased size of all three components was observed in metastasis-free LNs of metastatic patients.

### Model selection and experimental design

From 73 studies included in this review, 78% (*n*=57) were designed as patch classification experiments, whereas the remaining 22% (*n*=16) used pixel-level classification. In total, we found 27 distinct model architectures with 23 models describing patch classification and the remaining 4 models describing pixel-level segmentation studies. Among patch classifiers, ResNet models were utilized most frequently (15 studies),^27^^,^^46^, ^47^, ^48^, ^49^, ^50^, ^51^, ^52^, ^53^, ^54^, ^55^, ^56^^,^^72^^,^^84^^,^^98^ followed by MIL (*n*=9),^31^^,^^41^^,^^77^, ^78^, ^79^, ^80^, ^81^, ^82^^,^^99^ VGG models (*n*=8),^36^^,^^37^^,^^44^^,^^46^^,^^51^^,^^84^^,^^100^^,^^101^ and others as shown in Fig. 4B. Amongst pixel-level classification methods, U-Net was the most commonly used (10 studies)^26^^,^^27^^,^^30^^,^^59^, ^60^, ^61^, ^62^^,^^71^^,^^96^^,^^102^ followed by DeepLabv3 (*n*=7),^28^^,^^29^^,^^62^, ^63^, ^64^, ^65^, ^66^ Mask-RCNN (*n*=1),^16^ and proprietary Visiopharm AI metastasis model^90^ as can be seen in Fig. 4B.

### Chronological comparison of data and AI strategy selection

From a chronological perspective, patch-level classifier models pre-date pixel-level segmentation models, with the latter being increasingly used in recent years. Due to the popularity of the Camelyon challenges, earlier publications almost exclusively relied on Camelyon16, Camelyon17, and Patch Camelyon datasets, whereas publications with in-house datasets are increasingly emerging, see Fig. 4C. With respect to the research topic, studies focusing on metastasis-free LN compartment analysis emerged later than studies analyzing LN metastasis. All the studies on metastasis-free LNs use in-house dataset with the exception of Bekkhus et al, where a publicly available immunofluorescence dataset was used, and Gamez Serna et al, where rat mandibular LN dataset was utilized for LN contouring, see Table 1.

## Discussion

There is a growing amount of evidence that AI methods are applicable for digital pathology. The launch of the Camelyon challenges has greatly accelerated the development of metastasis detection algorithms for LNs, with over 300 studies citing the original publication of the Camelyon17 dataset to this date. Consequentially, the publications offering deep learning solutions identified during this review of the literature were mostly focusing on LN metastasis detection. The majority of the published studies using the Camelyon datasets present novel deep learning model adaptations to improve metastasis detection without offering some biological interpretation of the model performance, in particular causes for false-positive or false-negative predictions. The models selected for metastasis identification in LNs included both patch classifiers and segmentation methods. ResNet models were most frequently used for patch classifiers, whereas U-Net models were most frequently selected for segmentation tasks. The majority of metastasis detection models were designed as patch classifiers (84%), suggesting that patch classifier output is sufficient to predict the LN status (presence or absence of metastasis), and that refined tumor contour predictions obtained with segmentation methods may not be necessary for LN metastasis detection. From the selected papers, the best model performance on the Camelyon17 dataset was reported by Wang et al,^66^ where a modified DeepLabv3+ model followed by DBSCAN clustering and XGBoost for pN stage prediction reached the weighted kappa score of 0.9632. However, the same study reported lower detection accuracy for ITCs. One of the options to reduce the difficulties of micrometastasis and ITC detection in LNs could be the use of AI to help the pathologist by highlighting the suspicious regions in the LN slide to the pathologist. Such a solution has been suggested by Google AI, developing the LYNA model designed specifically for pathological LN analysis.^69^ The LYNA model has been designed to assist the pathologist by highlighting the potential areas with metastatic tumor allowing the pathologist to make the final decision for the analyzed slide.^68^ In the same study, the LYNA-assisted workflow achieved a significantly higher sensitivity for micrometastasis detection compared to AI-unassisted H&E slide analysis by an experienced pathologist. To our knowledge, there’s only one commercial software tool for LN metastasis detection provided by Visiopharm that is certified as an in vitro diagnostic medical device (CE IVD). In a study by Challa et al,^90^ the use of Visiopharm metastasis algorithm resulted in a perfect tumor detection sensitivity of 100%, yet the reported precision was 41.5% suggesting a high rate of false-positive metastasis predictions.

Despite the relatively large number of studies focusing on metastasis detection in LNs included in our review (63 papers), there are currently relatively few studies utilizing AI models to answer clinically driven research questions offering a biological interpretation of the model results and/or aiming at model implementation into the clinic. 12 studies analyzed the metastasis detection model performance in the biological context to characterize the falsely detected metastatic regions. They concluded that histiocyte-filled LN sinuses and germinal centers are the most common false-positive metastasis predictions in LN slides. The deep learning models enabled researchers to systematically assess metastasis dissemination in the LNs. Wang et al^27^ used a ResNet50 model to characterize the spatial patterns of metastatic spread in gastric cancer LNs, identifying two distinct mechanisms of metastasis based on the entry location of the tumor in the LN: via afferent lymphatic vessel or LN hilum. The authors trained a patch classifier at 20x magnification, which provided a sufficient output resolution to analyze the metastatic tumor location within LN achieving Dice score coefficient of 94.4%. To mitigate the putative false-positive predictions, the authors used LN sinus, germinal center, and adipose tissue patches as metastasis-free class during training. False-positive predictions in germinal centers could be tackled by developing a multi-class classification model that accounts for the germinal center class. This approach was implemented by Pham et al, who introduced a two-step deep learning pipeline combining two separate models for the detection of lymphoid follicles and metastasis regions.^44^

There is evidence accumulating to suggest that changes in secondary lymphoid follicles (germinal centers), sinuses, and paracortical areas of LN, so called reactive patterns (Fig. 1), may predict cancer patient survival. Allam et al^45^ described a method for pN stage prediction from non-metastatic part of metastatic LNs called tumor microenvironment, indicating that interfollicular lymphocyte areas have the highest positive-predictive power for breast cancer LN cases. We previously summarized reactive pattern evaluation protocols suggested so far for manual assessment of reactive LN status, which highlighted the need for a standardized guideline for LN reaction assessment.^103^ However, to establish evidence-based guidelines for LN reactive status evaluation, large scale biological studies need to be conducted to demonstrate its clinical value raising the necessity for automated detection methods. In this review, we identified six studies dedicated to AI-driven metastasis-free LN compartment analysis. The earliest study was published in 2019, highlighting the recency of this topic.^95^ The LN sinuses tend to have a complex shape of canals penetrating LN cross-section in various directions, which can be harder to segment using a patch classification approach at lower magnification. This could be concluded from the study by Jin et al,^97^ where the authors trained LYNA models for LN compartment detection at different magnifications. The highest accuracy for LN sinus segmentation was achieved analyzing patches at 40x magnification, whereas 10x magnification was optimal for breast cancer metastasis patch identification. In the same study, veins, arteries, nerves, LN capsule, fat, and lymphocytes were also best detected at 40x magnification except for germinal centers, where best test performance was achieved at 20x magnification. Manual LN sinus annotation proved to be a challenging task as suggested by Kurian et al, where authors implemented a U-Net autoencoder model for LN sinus segmentation accounting for noisy LN sinus annotations by developing a loss function for fuzzy boundaries.^96^ In a follow-up study from the same research group, the authors also explored the post-processing of the deep learning model output and identified evaluation metrics that could be translated into the clinical practice. The germinal center predictions were used to obtain metrics such as germinal center count per LN, germinal center mean area and germinal center circularity using the Polsby-Popper circularity metric for shape evaluation. For LN sinuses, the subcapsular sinus width per LN was evaluated. The study revealed that a larger number of germinal centers, a larger germinal center area, and its rounder shape, as well as wider subcapsular LN sinuses were associated with longer survival. However, these results require validation in studies in LNs from other types of cancer. In a recent study by Song et al,^98^ authors indicated the importance of prognostic LN structure size evaluation, concluding that the size of adipocytes, red blood cell conglomerates, and white gaps in LN have a prognostic significance when distinguishing patients by pN status and obesity stage. There was only a single study where the segmentation of HEVs was attempted. Bekkhus et al^16^ utilized a deep learning model to segment HEVs in immunofluorescent LN images. Besides HEV detection, the authors also aimed at HEV dilatation detection, as it was previously suggested that this might be associated with pre-metastatic changes in regional LNs.^104^ Further analysis is needed to validate whether HEV detection and dilation evaluation can be assessed in routine H&E-stained slides.

When designing an AI study, the selection of the deep learning model plays a fundamental role. The majority of the studies included in this review describe an adaptation of already existing model architectures for the LN analysis task and the most commonly used models are ResNet, DenseNet, and U-Net. The LYNA model introduced by Google AI was specifically designed to tackle the metastatic LN identification task, which was later adapted for metastasis-free LN compartment segmentation.^69^^,^^70^^,^^97^ The authors of LYNA model applied the multi-scale model paradigm, where the model accepts multiple input images at different magnifications, encompassing both low- and high magnification level features, to the LN analysis field. The multi-scale LN model idea was further developed by Schmitz et al,^71^ proposing a combination of an U-Net autoencoder and ResNet18 context encoder models. Gamez Serna et al^24^ introduced multi-scale MMO-Net model capable of multi-organ segmentation, including LNs. In a recent study by D’Amato et al,^105^ multi-scale models were proven to achieve higher F1 score metrics for tumor detection in 20 different cancer types compared to single-magnification setting, however the input–output intensive operations render multi-scale models timewise inefficient at WSI processing. The time efficiency issue has been addressed by Wang et al,^72^ proposing a model that reduced slide processing time more than eight times compared to LYNA model.

Dataset selection is another key factor when designing the computational study. Except for MMO-Net dataset released for rat mandibular LN contour detection, all the public datasets included in our review were exclusively generated from breast cancer LN cases with the ground truth specifically oriented towards metastasis evaluation (except HEV immunofluorescence dataset), which limits the scope of their usability for other research objectives. There is a need for alternative data sources. The low incidence of external validation of models found in the selected study pool (29%, *N*=21) is most likely related to the apparent lack of publicly available data that could be used as a ground truth in external validation. The Camelyon17 testing dataset contains slides from multiple different medical centers, addressing the practical challenge of site-specific WSI features, however, the slides originate from a single country (Netherlands), thus limiting the capabilities to evaluate model generalizability over different patient geographies.

The datasets used in the studies had a varying degree of public accessibility. Several datasets, such as OE02 esophageal cancer trial data as well as breast cancer LN dataset from Guy’s hospital had only images publicly available with inaccessible annotations, whereas only the datasets with accessible ground truth were regarded as publicly available in our review. The datasets varied in the nature of ground truth format—four datasets contained manually annotated ground truth, whereas AIDA breast LN dataset was complemented with immunohistochemically stained slides for metastasis. Compared to the manual annotations, immunohistochemically stained ground truth offers the highest annotation recall, as this method avoids the bias of human-produced errors. However, artifacts such as IHC stain residues might obfuscate the slide translation into ground truth mask, raising the need for pathologist to review the stained slides.^94^ Additionally, not all the anatomical structures of interest have a specific antibody that would produce annotation-quality staining. For instance, LN sinus macrophages have a specific stain for CD169+ macrophage cells, however, only a small subset of sinus-residing macrophages express CD169 molecule, whereas CD68 marker for macrophages is not specific for this cell type only,^106^ which would produce false positive staining. Due to these issues, the ground truth annotation process requires an expert-in-a-loop to ensure reliable outcome. However, the laborious annotation process has a potential to be at least partially automated by applying novel annotation tools that implement active learning, such as Monai Label, DeePathology, or DigiPath Viewer.

### Limitations

With the exception of one study,^16^ studies included in this review were limited to those using light microscopy imaging data because the primary focus of this review was the analysis of routine H&E-stained slides. However, numerous studies have been performed on other sample analysis methods. Miura et al^107^ have employed scanning acoustic microscopy to identify lesion elasticity in LNs. Liu et al^108^ relied on multi-spectral microscopy to obtain elliptic features of cells, enabling LN segmentation into tumor, B, T, and dendritic cells. Fourier transform infrared (FTIR) microscopy has been applied for LN architecture analysis, leading to complete unsupervised LN architecture reconstruction by Bird et al,^109^ followed by later study by Leslie et al^110^ rendering higher-resolution LN microarchitecture features of eight distinct classes. Compared to light microscopy, each analysis method has specific advantages: scanning acoustic microscopy is capable of analyzing tissue elasticity, multi-spectral microscopy includes spectral bands in infrared and ultraviolet wavelengths besides red, green, and blue color channels, FTIR method captures cell-specific molecular composition, thus offering higher resolution than H&E stained tissue analysis. These methods, despite their own limitations, could complement the traditional H&E sample analysis by adding new insights into tissue composition and biochemical processes inside LNs.

This review was limited to the studies analyzing LNs without further investigating the structural composition of the primary tumor. Tertiary lymphoid structures (TLSs) found inside and nearby the primary tumor are gaining scientific attention. In the study by Ling et al,^111^ Inception-ResNet-v2 model was trained for patch classification to detect TLSs in esophageal cancer patients and to distinguish between immature and mature TLS cases. In the same study, mature TLSs have been associated with a better patient disease-free survival. Barmpoutis et al^112^ have published their approach for TLS segmentation in lung cancer patients using DeepLabv3+ and Inception-ResNet-v2 for feature extraction.

The heterogeneity of datasets and their usage in the studies have limited our capabilities to perform a meta-analysis on the included studies, limiting the quantitative power of study comparison. For the metastasis detection task, 68% (*n*=43) of all included metastasis detection studies used one or more of five publicly available datasets, whereas 39% (*n*=25) of studies employed inhouse, publicly inaccessible datasets with or without mixing it with publicly available data. In case of metastasis-free LN analysis, five out of six reviewed studies used different publicly unavailable datasets. We considered that quantitative AI method comparison for models trained and tested on different datasets would carry a limited value, thus we refrained from quantitative data interpretation across the reviewed articles. Another limitation of this study was the language, as only the articles published in English were included, thus constricting the range of articles screened for our analysis.

## Conclusions

Based on the published evidence gathered for this review, it can be concluded that the computational lymph node analysis field is currently evolving. The LN metastasis detection task has been extensively explored by the scientific community, leading to promising commercial solutions such as Visiopharm metastasis app, Paige Breast Lymph Node system. However, when considering clinical implementation, challenges such as sensitivity and specificity maximization, as well as model robustness evaluation via external testing sets need to be addressed. These practical challenges highlight the current limitations of accessible annotated LN datasets. New research studies are emerging acknowledging the computational characterization of metastasis-free LN compartment rearrangements, resulting in first attempts to segment LN compartments in H&E-stained slides including LN sinuses, germinal centers, vessels, and paracortical lymphocyte areas. Further research is warranted to establish and validate the clinically interpretable metrics describing the features of segmented LN structures.

## Declaration of competing interest

The authors declare the following financial interests/personal relationships which may be considered as potential competing interests:

H.W. has minority shares in the company Radiomics SA. D.R.M. is a director of HeteroGenius Limited. If there are other authors, they declare that they have no known competing financial interests or personal relationships that could have appeared to influence the work reported in this paper.
