# Corrigendum to “Computational methods for metastasis detection in lymph nodes and characterization of the metastasis-free lymph node microarchitecture: A systematic-narrative hybrid review”. *Journal of Pathology Informatics* 15(2024) 100367

**DOI:** 10.1016/j.jpi.2025.100457

**Published:** 2025-07-07

**Authors:** Elzbieta Budginaite, Derek R. Magee, Maximilian Kloft, Henry C. Woodruff, Heike I. Grabsch

**Affiliations:** a Department of Pathology, GROW - Research Institute for Oncology and Reproduction, Maastricht University Medical Center+, Maastricht, the Netherlands; b School of Computing, University of Leeds, Leeds, UK; c Department of Internal Medicine, Justus-Liebig-University, Giessen, Germany; d Department of Precision Medicine, GROW - Research Institute for Oncology and Reproduction, Maastricht University Medical Center+, Maastricht, the Netherlands; e Pathology and Data Analytics, Leeds Institute of Medical Research at St James's, University of Leeds, Leeds, UK

The authors regret to inform that the Acknowledgments section of the manuscript is missing funding information. The section should begin with the following sentence, followed by the existing acknowledgments:

“This research was funded by the Hanarth Fonds.”

The authors would like to apologize for any inconvenience caused.

