# Supplementary material for: Computational methods for metastasis detection in lymph nodes and characterization of the metastasis-free lymph node microarchitecture: A systematic-narrative hybrid review
Source: J Pathol Inform. 2024 Feb 4;15:100367. doi: 10.1016/j.jpi.2024.100367 (PMC10918266; doi:10.1016/j.jpi.2024.100367)
Supplement: Supplementary material 1 [file mmc1.docx]

Computational methods for metastasis detection in lymph nodes and characterization of the metastasis-free lymph node microarchitecture: A systematic-narrative hybrid review

Supplementary Table S1 Search phrases used across databases for article identification.

| Search phrases |
| --- |
| lymph node AND artificial intelligence AND histology OR pathology;  lymph node AND deep learning AND histology OR pathology;  digital pathology AND spatial;  lymph node AND machine learning AND histology OR pathology;  lymph node AND segmentation. |

Supplementary Table S3 Extracted parameters from the selected studies describing the study objective, used AI model and its documented performance, cancer type and dataset selection and lymph node slide magnification used for training.

| Author (year) | LN analysis topic | Magnification | Cancer type | Dataset(s) | AI model | Performance results |
| --- | --- | --- | --- | --- | --- | --- |
| Niemisto (2005) | contours | NA | Colorectal | Inhouse dataset, where single image contains multiple pathology slides | k-means | Manual vs. automated lymph node count correlation |
| Wang (2016) | metastasis | 40x | Breast | Camelyon16 | GoogleNet, AlexNet, VGG16, FaceNet, binary heatmap for lesion detection | AUC: 0.925; FROC: 0.7051 |
| Jamaluddin (2017) | metastasis | NA | Breast | Camelyon16 | AlexNet, GoogleNet, VGG16. Random forest classifier for whole slide classification task, tumor probability score | AUC 0.94 |
| Liu (2017) | metastasis | 40x | Breast | Camelyon16, patches of lnneg vs metastatic. NHO-1 dataset for external validation | LYNA model original release, LYNA patch classifier incorporating Inception v3 architecture in multi-scale manner | AUC 97.7%; FROC: 88.5% |
| Valkonen (2017) | metastasis | NA | Breast | Camelyon16 | NN, LR, SVM, random forest on hand-crafted features for patch classification | AUC: 0.97-0.98 |
| Akbar (2018) | metastasis | 10x | Breast | Camelyon16 | Bag-level MIL, cluster based, latent representations for VAE; categorical cross-entropy loss modification for k-means clustering result | Accuracy: 97.91% |
| Lee and Paeng (2018) | metastasis | 40x | Breast | Camelyon17/16 | ResNet101 patch classifier for metastasis detection, random forest classifier for pN prediction from hand-crafted features from mask | Quadratic weighted kappa: 0.9203; AUC: 0.985; FROC: 0.955 |
| Lin (2018) | metastasis | NA | Breast | Camelyon16 | FCN-based ScanNet, fast metastasis localization | AUC: 0.9875; FROC: 0.8533; Efficacy (minute): 0.018 |
| Steiner (2018) | metastasis | NA | Breast | Inhouse dataset from clinical partners | LYNA model | Average review time(s):111; Sensitivity: 91.2% |
| Veeling (2018) | metastasis | 10x | Breast | Patch Camelyon | CNN with rotation equivariance, patch Camelyon dataset introduced | Accuracy: 89.8%; AUC: 96.3%; FROC: 84% |
| Wollmann (2018) | metastasis | NA | Breast | Camelyon17 | CycleGAN for image augmentations; tile classification with DenseNet model into 4 classes: ITC, micro, macro metastasis, non-metastatic | Weighted Cohen’s kappa: 0.165 |
| Alheejawi (2019) | metastasis | NA | Melanoma | Inohuse IHC stained dataset | SegNet vs FCN, GMM, Otsu/SVM | Accuracy: 95.55%; Specificity: 93.15%; Sensitivity: 94.81%; Precision: 90.98% |
| Jaiswal (2019) | metastasis | 10x | Breast | Patch Camelyon | Semi-supervised approach for VGG16, Inception-ResNetv2, Inception, Xception, ResNet101, DenseNet101. Test time augmentations for model performance improvement | AUC: 0.9786 |
| Kovalev (2019) | metastasis | NA | Breast | Camelyon16 | DCGAN and PGGAN for augmentation; models for classification: VGG16, KNN, SVM, Random forest | Accuracy: 0.93% |
| Lin (2019) | metastasis | NA | Breast | Camelyon16 | FastScanNet: anchor layers for scannet based on VGG16. Anchors compute missing patches | AUC: 0.9875; FROC: 0.8533; Efficacy (minute): 0.0182 |
| Liu (2019) | metastasis | 40x | Breast | Inhouse dataset excluding IITC cases | LYNA model (lymph node assistant), false positive analysis, color normalization for validation dataset | AUC: 99%; Sensitivity: 91%; External test set AUC: 99.6% |
| Palatnik de Sousa (2019) | metastasis | NA | Breast | Patch Camelyon | SLIC superpixels, LIME | AUC 0.9683,; Result interpreted from explainability maps |
| Pham (2019) | metastasis | step1: follicle detection at 1MPP (10x), step2: tumor cell detection at 40x | Lung | Inhouse dataset from japanese hospital | HALO software for annotation/ training/ classification. Random forest and VGG networks for tissue classification: macromestastasis, micrometastasis, ITC, nonmetastatic. | Accuracy 89% ; Sensitivity: 79.6%; Specificity: 96.5% |
| Qaiser (2019) | ECM | NA | Diffuse large B-cell lymphoma | Inhouse dataset | Hydra-net for cell classification; k-means clustering for collagen-tumor proximity evaluation (weak, moderate, significant and strong association between collagen and tumor areas) | Cell classification: F1 Score: 0.84; Precision: 0.839; Recall: 0.842 |
| Xu (2019) | metastasis | 20x | Breast | Camelyon16, Camelyon17 | U-Net, patch based segmentation; hand-crafted features subjected to random forest classifier for macro/micro/itc/negative LN classification | Kappa score: 0.902 |
| Zhang (2019) | metastasis | 40x | Breast | Camelyon16 | CNN with spatially fusing patches: multiple spatial context network (MSC-net); patch feature sharing for faster slide scanning | Accuracy: 98.43%; FROC: 0.8078 |
| Bozdag (2020) | metastasis | 3rd level magn | Breast | Camelyon16 | Asymmetric pyramid nonclonal block + DeepLabv3, SegNet, pspNet | Accuracy: 85.1%; Mean IoU: 68.6% |
| Cheng (2020) | metastasis | NA | Breast | Camelyon16 and inhouse taiwanese hospital wsis | Patch classifier student-teacher model setup; self-sim student for noisy label learning | DSC: 93.76%; FROC: 36.90% |
| Courtiol (2020) | metastasis | NA | Breast | Camelyon16 | Weakly supervised CNN using global labels; Weldon method: MIL approach for top instances and negative evidence | AUC: 0.8706 |
| Gildenblat (2020) | metastasis | NA | Breast | Camelyon16 | Siamese network leveraging spatial continuity, assuming spatially adjacent tiles are more similar than distant tiles | Average descriptor distance ratio (ADDR): 1.5; Ratio of retrieved tumor tiles: 35% |
| Jiarong (2020) | metastasis | NA | Breast | Camelyon16 | HistoGan; ResNet34 backbone | Accuracy: 0.835; AUC: 0.89; Sensitivity: 0.747; Specificity: 0.936 |
| Jin (2020) | metastasis | 40x | Breast | Inhouse dataset for tubules, mitosis, nuclei masks , Patch Camelyon | 4 U-Net models concatenated, ConcatNet; integrated gradients algorithm for visualizing interpretations of the same input between different models | AUC: 0.924; Sensitivity: 82.0 %; Specificity: 87.8% |
| Kim (2020) | metastasis | NA | Breast | Inhouse datasets from Seoul hospitals; for pre-training stage: Camelyon16 | Inceptionv3, VGG16, ResNet | AUC: 0.944; External AUC: 0.844 |
| Pan (2020) | metastasis | NA | Esophageal | Inhouse dataset from chinese hospital including clinical variables | DeepLabv3 on ResNet50 backbone; DeepLabv2 + cross-entropy loss, DeepLabv3 + cross-entropy loss, DeepLabv3 + focal loss | Accuracy: 94%; AUC 0.96 |
| Chuang (2021) | metastasis | 4x | Colorectal | Inhouse dataset from Taiwanese hospital | ResNet50, CAM for morphologic feature identification in tumor detection | Accuracy: 98.5%; AUC: 0.9957 |
| Hu (2021) | metastasis | NA | Gastric | Inhouse dataset | Cascade algoritm of Faster RCNN + DeepLabv3 for LN object detection, patch-based classification on Xception and DenseNet121 model features merged with global maxpooling | Accuracy: 97.13%; PPV: 93.53%; NPV: 97.99% |
| Luz (2021) | metastasis | NA | Breast | Patch Camelyon | LeNet, VGG16, VGG19/ SVM, random forest classifier using pre-trained NN models | Accuracy: 83.63%; Specificity: 72.49%; Sensitivity: 94.76%; AUC: 93% |
| Schmitz (2021) | metastasis | 1x, 4x, 16x | Breast | Camelyon16 | Multi-scale U-Net | Jaccard index: 0.859 |
| Shao (2021) | metastasis | NA | Breast | Camelyon16 | TransMIL: transformer based MIL model (patch classifier); balanced/unbalanced dataset cases; attention heatmap for explainability | Accuracy: 88.37% ; AUC: 93.09% |
| Shubin (2021) | metastasis | NA | Breast | Patch Camelyon | Variance-aware; self-supervised | AUC: 0.806 |
| Shvetsova (2021) | metastasis | NA | Breast | Camelyon17 | Autoencoder with perceptual loss for anomaly detection in PCam dataset. Model computes the distance between deep features from object classification NN pretrained on diverse dataset. | AUC: 93.4% |
| Turki (2021) | metastasis | NA | Breast | H&E stained slides containing axillary LNs from the cancer imaging archive | ResNet, DenseNet121, VGG16, Xception | AUC: 64-68% |
| Wang (2021) | metastasis | NA | Breast | Camelyon16, external dataset from thomas fuchs lab at TCIA | Second-order MIL: tile level classifier with ResNet backbone | Accuracy: 0.936; AUC: 0.935 |
| Wang (2021) | metastasis | NA | Breast | Camelyon17 | Coarse metastasis prediction using DSNet, dilated ResNet101 model with ASPP; DBSCAN clustering to fuse patch predictions into slide level prediction(spatially adjacent metastases). XGBoost for pN stage classification. Compared with DeepLab pipeline | Cohen's Kappa Score: 0.9473; weighted kappa score: 0.9632 |
| Wang (2021) | metastasis | 20x | Gastric | Inhouse dataset from chinese hospital | LN contour detection: 1x magnification U-Net, patch classifier: ResNet50 | Segmentation:; DSC: 98.6%; Mean Jaccard index: 95.8%; Classification:; AUC: 0.99; DSC: 94.4%; FROC: 0.872 |
| Xue (2021) | metastasis | NA | Cervical, breast | Inhouse dataset from collaborating health sciences center for cervical cancer; breast cancer: Pacth Camelyon | HistoGAN for synthetic image augmentation | Accuracy: 0.821; AUC: 0.881; Sensitivity: 0.671; Specificity: 0.917 |
| Abdollahi (2022) | metastasis | NA | Breast | Patch Camelyon | Ensemble CNNs: VGG16, ResNet50, MobileNet, GoogleNet | Accuracy: 98.84 % ; AUC: 96.02%; Precision: 92.42%; Recall: 91.25% |
| Bekkhus (2022) | substructures | NA | Breast | Immunofluorescence dataset | Mask-RCNN | Accuracy: 68.5% |
| Chen (2022) | metastasis | NA | Breast | Camelyon16 + inhouse datset | Xception, Inception, ResNet, RRCART | Accuracy (high-accuracy group): 0.995 |
| Gamez Serna (2022) | contours | 1.25x; 5x | Mandibular LNs | MMO-Net publicly available dataset | HALO software for annotations; DenseNet121 model on 1.25 and 5x magnifications, PyTorch framework. One-vs-all binary models | AUROC: 1; DSC: 0.95 |
| Huang (2022) | metastasis | 20x | Gastric | Inhouse dataset of HE and IHC-stained LN samples from chinese hospital | Weakly supervised enhanced straing CNN, ResNet50. For LN detection: DeepLabv3 model | Sensitivity: 0.8915; Specificity: 0.9861; Mattews correlation coefficient: 0.8986; Sensitivity (micrometastasis): 95.83%; Sensitivity (isolated tumor cells): 96.15%; Review time: -31%; External AUC: 0.9829 |
| Jarkman (2022) | metastasis | NA | Breast | Camelyon17, Camelyon16, AIDA BRLN dataset | DenseNet classifier | AUC: 0.969; FROC: 0.838; External data AUC: 0.929; FROC: 0.744 |
| Jin (2022) | substructures | 10x, 20x, 40x | Breast | Inhouse dataset from US hospital, available upon request. Annotated by 3 pathologists | LYNA model | AUC: 0.98; Accuracy: 0.94; Sensitivity: 0.88 |
| Khalil (2022) | metastasis | NA | Breast | Inhouse dataset of H&E and coresponding ihc for cytokeratin from taiwanese hospital | DCNN, Xception, customized FCN model, DeepLabv3 using MobileNet, ResNet or Xception, UNet, SegNet | Precision: 89.6%; Precision: 83.6%; F1 score: 84.4%; mIoU: 74.9% |
| Khan (2022) | external validation | 20x | Colorectal | Inhouse datset from swiss hospitals | U-Net with domain adversarial learning and stain mix-up augmentation to account for scanner variability | Mattews correlation coefficient: 0.87 |
| Kronberg (2022) | metastasis | NA | Pancreatic ductal | Inhouse dataset, 20x and 40x scanned TMAs | ResNet18 | Accuracy: 0.94%; Precision: 0.94; Recall: 0.94; F1-Score: 0.94; Jaccard score: 0.89 |
| Kurian (2022) | substructures | NA | Unk | Inhouse dataset | U-Net with multi-scale and attention mechanism adaptations | IoU Germinal centers: 0.7842; IoU Sinus: 0.6047 |
| Mainovskaya (2022) | metastasis | NA | Colorectal | Inhouse dataset from russian hospital for artifact and crc metastasis | DeepLabv3 + U-Net | Substituted tissue DSC: 0.863; Artefact DSC: 0.552 |
| Matsushima (2022) | metastasis | NA | Gastric | Inhouse GC samples, HE stained and IHC counter-stained | ResNet-152 | AUC: 0.9994; Sensitivity: 1.00 |
| Palatnik de Sousa (2022) | metastasis | NA | Breast | Patch Camelyon | LIME, SLIC for superpixels. EvEx algorithm to generate explanations via multi-objective genetic algorithm | Relative standard deviation (RSD) for reproducibility and Explanation score (Es), Result interpreted from explainability maps |
| Tang (2022) | metastasis | 10x | Head and neck squamous carcinoma | Head and neck scc inhouse dataset from chinese hospital | GoogleNet, MobileNetv2, ResNet50, ResNet101 | Accuracy: 86%; Sensitivity: 100%; Specificity: 75.9% |
| Wang (2022) | metastasis | NA | Breast | Camelyon16 | MIL, label cleaning for bag classification | F1 score: 0.882; PPV: 0.863; TPR: 0.915 |
| Zhao (2022) | metastasis | NA | Breast | Camelyon16 | RL+CLAM. RL: Markov decision process (MDP); tile classification | Accuracy: 0.8685; AUC: 0.8874; Average runtime (min): 9.74 |
| Allam (2023) | metastasis | NA | Breast | Inhouse | CNN model, custom architecture | Accuracy 91.15%; Sensitivity: 77.92%; Specificity: 92.09%; PPV:90.86%; NPV: 80,66% |
| Beuque (2023) | contours | thumbnails | Esophageal | OE02, inhouse annotations | U-Net for contours, XGBoost for false positive exclusion on radiomics features | Balanced accuracy: 0.93; Dice score per-slide 0.73; Dice score per-LN: 0.66 |
| Challa (2023) | metastasis | 1x for tissue detection, unspecified for metastasis | Breast | 3 inhouse datasets, 2 containing SNLs, 1 on-sentinel LNs | Visiopharm integrator system metastasis AI algorithm | Sensitivity: 100%; Specificity: 41.5%; PPV: 29.5%; NPV: 100% |
| Jansen (2023) | metastasis | 20x | Melanoma | Inhouse from 4 medical centers (2 for testing) | Resnet-U-Net | AUC: 0.96 and 0.98 for 2 cohorts |
| Kang (2023) | metastasis | NA | Esophagral | Inhouse from 2 hospitals and Camelyon 16/17 | ResNet50 + MIL and transfer learning | Accuracy: 0.976 (0.969 external); AUC: 0.991 (0.988 external); F1-score: 0.944 (0.925 extrenal), Mattews correlation coefficient: 0.929 (0.905 external) |
| Matsushima (2023) | metastasis | NA | Gastric | Inhouse from 2 centers | ResNet152 | AUC (metastasis): 0.869; AUC (micrometastasis): 0.785 |
| Munappa (2023) | metastasis | NA | Breast | Camelyon17 | Ensemble of pre-trained ResNet50, EficcientNetB3, DenseNet121 models | Accuracy 0,966; AUC: 0.958; Precision: 0.85; Recall: 0.91 |
| Patil (2023) | metastasis | 10x | Breast | Camelyon16 and publicly available HistoROI dataset of 6 classes | HistoROI (ResNet16 model) | AUC 0.9 |
| Qin (2023) | metastasis | NA | Breast | Camelyon16 | Pathology transformer (PathTR) | FROC: 87.68% |
| Sadafi (2023) | metastasis | NA | Breast | Camelyon17 | Attention-based MIL with active learning | Accuracy: 0.72; AUC: 0.64; F1-score: 0.69 |
| Song (2023) | substructures | NA | Breast | Inhouse | Rule based algorithms for adipocyte, lymphoid white space and red blood cell detection. Deep learning model for patient-level class assignment | AUC: 0.67 |
| Tan (2023) | metastasis | NS | Colorectal | Inhouse | Transformer-based MIL model for classification, Faster-RCNN for lymph node identification | Accuracy: 95.3%; AUC: 0.976 |
| Verghese (2023) | substructures | 10x | Breast | Inhouse | Multi-scale U-Net | DSC (germinal centers): 0.86; DSC (sinuses): 0.74 |
| Wang (2023) | metastasis | 10x and 40x | Breast | Camelyon16 | Multi-scale model based on ResNet18 | FROC: 0.871 |
| Yu (2023) | metastasis | NA | Breast | Camelyon16 and inhouse | Prototypical MIL | AUC: 88.2% |
